# From static supply to time-weighted path-based dynamic blue-green exposure: spatial mismatches between blue-green environments and platform-recorded walking use in Tianjin

**DOI:** 10.3389/fpubh.2026.1967963

**Published:** 2026-10-08

**Authors:** Xiaohan Jia, Yujia Chen

**Affiliations:** 1 School of Architecture and Art Design, Lanzhou University of Technology, Lanzhou, China; 2 China School of Architecture, Tianjin University, Tianjin, China

**Keywords:** blue-green space, SHAP, spatial distribution, time-weighted path-based dynamic blue-green exposure, walking use

## Abstract

Assessments based only on blue-green resource supply within fixed spatial units may not capture environmental conditions encountered along movement routes or their spatial co-occurrence with platform-recorded use. Using Tianjin as the study area, we integrate publicly shared Keep walking trajectories with street-view imagery, remote-sensing imagery, environmental stress, and socioeconomic data. At the grid level, we measure static blue-green supply (SBG), time-weighted path-based dynamic blue-green exposure (DBG), environmental stress (ES), and Keep walking activity density (WAD). We then apply population-weighted Gini coefficients, quadrant-based mismatch analysis, a composite Priority Index (PI), and interpretable machine-learning methods with spatial cross-validation. Population-weighted Gini coefficients were 0.448 for SBG, 0.331 for DBG, and 0.374 for ES, indicating different degrees of distributional concentration relative to population. The low SBG-high WAD, low DBG-high WAD, and high ES-high WAD classes overlapped only partly, indicating distinct spatial patterns among resource supply, reconstructed-route exposure, environmental burden, and platform-recorded use. All four predictor domains provided predictive information, while out-of-fold SHAP patterns suggested broad nonlinear predictive associations with apparent turning points for several variables. Random Forest performance under spatial five-fold cross-validation was nevertheless limited ( $R^{2}$ = 0.051 ± 0.027), so these model-based patterns are treated as exploratory and complementary to the main spatial mismatch analysis. Static resource supply and reconstructed-route DBG therefore represent distinct environmental dimensions. Taken together, these measures provide a descriptive approach for distinguishing forms of environment-use mismatch and screening candidate areas for subsequent field verification.

## 1 Introduction

Walking is a basic form of daily physical activity for urban residents and an important mode of active transport that connects residential spaces, public-service facilities, and urban open spaces (1–3). Amid rapid urbanization, concern has grown about sedentary behavior, traffic congestion, air pollution, and heat exposure (4). Promoting daily walking has consequently become an objective of healthy-city and sustainable urban planning (5, 6). Research links both the decision to walk and the spatial distribution of walking to individual characteristics and built-environment factors, including street connectivity, land-use mix, destination accessibility, population density, walking infrastructure, and environmental quality (2, 7–9). Urban planning, public health, and environmental equity research therefore examines how spatial environments support walking and whether that support is equitably distributed across places and populations (2, 10).

Blue-green spaces are an important component of urban natural infrastructure. Observational research has associated blue-green environments with walking and physical activity through attributes such as shade and thermal comfort, air quality, visual quality, recreational opportunities, and psychological restoration (5, 11–13). Most of this evidence is observational, so causal inference remains limited. Green spaces and water bodies are also often distributed unevenly (10, 14–16). Their supply may not align with population needs, accessibility, environmental burdens, or observed use (17–20). Measures based only on total resource quantity or area-average levels can therefore obscure both allocation relative to population and the spatial relationship between environmental conditions and use. Recent research has begun to compare resource supply, exposure, environmental burden, and use within integrated frameworks (14, 20, 21).

Most assessments of blue-green environments still rely on fixed spatial units, such as land-use parcels and residential buffers, or on remotely sensed measures. These approaches describe static resource supply at a location but capture less well the environmental conditions encountered while moving (22–24). Location-based services and trajectory data allow process-based exposure to be approximated from walking routes and segment-level travel times (7, 23, 25, 26). Here, time-weighted path-based dynamic blue-green exposure (DBG) refers to route-based exposure estimated from API-reconstructed walking routes by weighting environmental conditions according to segment-level walking time; it does not refer to change across years or time points. Because these routes are proxies rather than exact reproductions of recorded Keep trajectories, DBG represents reconstructed-route exposure rather than the actual individual exposure of Keep users. DBG may also reflect route-choice behavior and is therefore interpreted at the grid level (22, 23, 26). Publicly shared platform trajectories likewise cover only users who use the platform and choose to share their records; they do not represent the daily walking of all urban residents (5, 27).

Associations between the built environment and walking activity are often nonlinear and may include thresholds (28–31). A single linear model may therefore fail to capture these patterns and interactions (32). Ensemble methods such as Random Forest and XGBoost can fit these complex relationships, while SHAP can compare variables’ relative predictive contributions and the directions of their associations (33–35). Because urban data often exhibit spatial dependence, random cross-validation alone may overstate generalizability to unobserved areas. We therefore compare random and spatial cross-validation and include static blue-green supply, DBG, environmental stress, and socioeconomic and locational factors in one model. Rather than favoring a particular algorithm, the analysis compares the relative contributions and nonlinear associations of environmental dimensions under spatial validation. These comparisons directly address questions about spatial heterogeneity in urban environments and walking activity. Machine-learning analysis complements the spatial mismatch analysis by describing relative predictive contributions and broad nonlinear patterns.

The literature leaves three related gaps. Most blue-green environment assessments focus on static resource supply within fixed spatial units rather than route-based exposure during movement (16, 36, 37). Environmental equity studies seldom examine spatial mismatches among static supply, DBG, environmental stress, and platform-recorded walking use in a single framework. Direct comparisons of the relative predictive contributions, nonlinear associations, and spatial generalizability of static blue-green and DBG predictors also remain limited (29, 31). A common descriptive and methodological framework is therefore needed to examine resource supply, reconstructed-route exposure, environmental burden, and platform-recorded use together.

Using Tianjin as a case study, we combine Keep walking trajectories, street-view imagery, remote-sensing imagery, environmental stress, and socioeconomic data in a multi-source data-fusion framework. The analysis addresses three questions: how static blue-green supply, DBG, and environmental stress are distributed relative to population; where static supply, reconstructed-route DBG, environmental stress, and platform-recorded walking use co-occur or diverge spatially; and what relative predictive information the corresponding environmental and socioeconomic domains provide under spatial validation. Population-weighted Gini coefficients compare distributional inequality in SBG, DBG, and ES, while quadrant classification and a composite Priority Index (PI) provide descriptive screening of candidate mismatch areas. The study makes a descriptive and methodological contribution in three respects. First, it distinguishes fixed-grid blue-green resource supply from reconstructed-route exposure as related but non-equivalent environmental constructs. Second, it places static supply, reconstructed-route exposure, environmental stress, and platform-recorded walking use within a common spatial framework to identify where these dimensions co-occur or diverge. Third, spatial cross-validation and out-of-fold model interpretation are used to examine whether the corresponding predictor domains retain predictive information beyond local spatial similarity.

## 2 Materials and methods

### 2.1 Study area and research framework

Tianjin lies on China’s northern coast and in the lower reaches of the Haihe River Basin. Its urban form includes a high-density central city, the Haihe waterfront corridor, coastal wetlands, port-industrial areas, and peripheral ecological spaces. The left panel of Figure 1 locates Tianjin within China; the right panel maps the study area’s administrative divisions, major roads, water bodies, and green spaces. Green space is relatively concentrated in northern Jizhou District, whereas water bodies are more extensive in the eastern and southeastern coastal areas. Road networks are denser and development intensity is higher in and around the central city. This mix of landscape and development types produces substantial spatial variation in blue-green environments and urban activity, making Tianjin suitable for comparing static resource supply with DBG (38).

**Figure 1:**
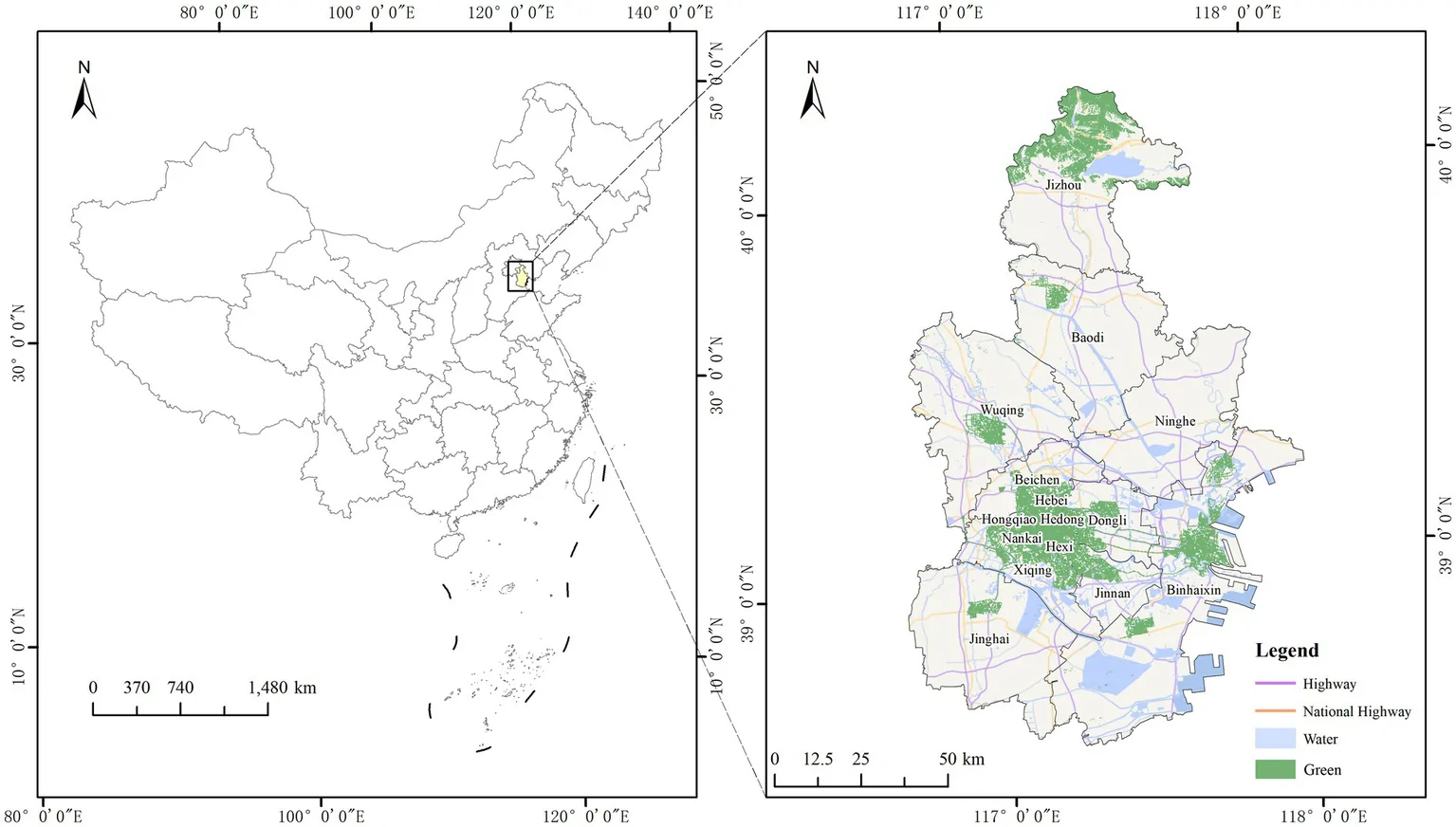
Location of Tianjin and the spatial distribution of major water bodies, green spaces, and transportation networks. The left panel shows the location of Tianjin in China, and the right panel shows blue-green spaces and major road networks within the study area.

Figure 2 summarizes three stages of the research workflow: data collection and preprocessing; indicator construction and grid-based integration; and spatial distribution and mismatch assessment, followed by modeling and interpretation. Keep walking trajectories, street-view imagery, remote-sensing imagery, environmental stress, and socioeconomic data are first integrated and standardized to 200 m × 200 m regular grids. We then construct Keep walking activity density (WAD), the static blue-green supply index (SBG), DBG, and the environmental stress index (ES). Population-weighted inequality in SBG, DBG, and ES is compared, and quadrant-based mismatch analysis and a composite Priority Index (PI) screen candidate grids where high platform-recorded use co-occurs with relatively low environmental indicator values or high environmental stress. Finally, Linear Regression, Random Forest, XGBoost, and LightGBM are compared using the same random and spatial folds. The selected model is interpreted with out-of-fold SHAP, grouped permutation, interaction diagnostics, residual spatial testing, and robustness analyses.

**Figure 2:**
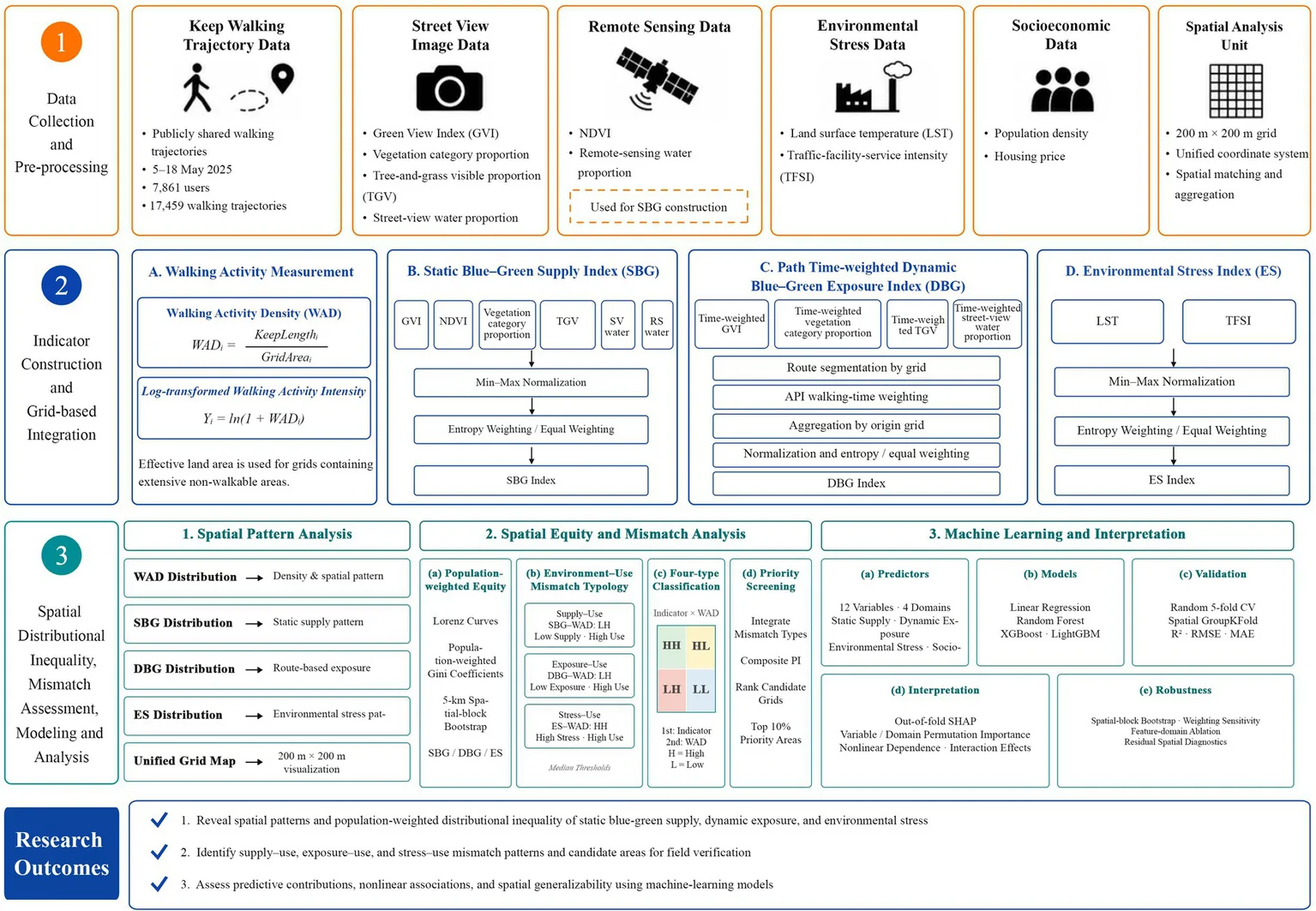
Research framework. The workflow includes multi-source data preprocessing, indicator construction and grid-based integration, spatial distribution and mismatch analysis, spatial cross-validation, model interpretation, and robustness testing.

The basic spatial unit is a 200 m × 200 m regular grid. Unlike administrative units, whose area and boundary shape vary substantially, regular grids provide more consistent areas and boundaries. This improves spatial comparability and reduces the influence of administrative-boundary differences on spatial statistics (39). Under a common coordinate system, all walking trajectories, street-view indicators, remote-sensing indicators, environmental stress variables, and socioeconomic variables are aggregated to the grid level (5, 39). Calculations use the actual area of grids intersected by the study-area boundary. For grids that contain large water bodies, wetlands, or other non-walkable spaces, walking activity density is adjusted using effective land area.

The 200 m resolution was selected as a practical compromise between pedestrian-scale spatial detail and multi-source data density: finer grids can increase sparsity in trajectory and street-view observations, whereas coarser resolutions such as 250–500 m may smooth local environmental variation relevant to walking.

### 2.2 Data sources and preprocessing

The study combines keep walking trajectories, street-view imagery, remote-sensing imagery, environmental stress, socioeconomic data, and basic geographic data. The Keep dataset comprises publicly shared platform records collected from May 5 to May 18, 2025: 14 days, 7,861 users, and 17,459 walking trajectories. Preprocessing removed duplicate records, checked abnormal coordinates and speeds, screened trajectory length and duration for plausibility, harmonized coordinate systems, and clipped the data to the study area (5, 40). The cleaned linear trajectories were intersected with the regular grid and segmented, after which segment lengths were summed within each grid. These records capture only spatial use among platform users who publicly shared their data, not the complete daily walking behavior of all residents. We therefore use the term “Keep walking use” for results derived from these records (27).

Street-view and remote-sensing data jointly characterize the static blue-green environment. The street-view indicators are the Green View Index (GVI), vegetation-category proportion, tree-and-grass visible proportion (TGV), and street-view water proportion; the remote-sensing indicators are the Normalized Difference Vegetation Index (NDVI) and remote-sensing water proportion. Street-view images were obtained from Baidu Street View. After matching images to sampling points, we checked image quality and performed semantic segmentation using Mask2Former. The segmentation outputs followed an ADE20K-style 150-class semantic taxonomy, and the model achieved a mean intersection over union (mIoU) of 62.7%. Green and blue features visible from a pedestrian perspective were then extracted (41–43). Static GVI was calculated as the summed visible-pixel proportion of the tree, grass, and plant classes. TGV was calculated as the summed visible-pixel proportion of the tree and grass classes. Street-view indicators were averaged across valid samples in each grid. For the remote-sensing imagery, we removed clouds and shadows, corrected bands, harmonized spatial resolution, and clipped the images to the study area. Each grid was then summarized by its mean pixel value or area proportion (44–47).

We estimate DBG along API-reconstructed walking routes. Routes were reconstructed using the Amap Web Service walking-direction endpoint. Requests were submitted between consecutive cleaned Keep trajectory points using only the key, origin, and destination parameters; no routing-strategy or waypoint parameter was specified. The first returned walking path was retained and its step polylines were concatenated. The resulting route geometry was intersected with the analytical grid, and its GVI, vegetation-category proportion, TGV, and street-view water proportion were weighted by the API-reported segment-level walking time. Mean exposure across valid reconstructed routes is then assigned to the route-origin grid (22, 23, 25, 26). DBG construction produced 4,725 assigned reconstructed routes for the route audit reported in Section 3.6. DBG therefore approximates process-based exposure along API-reconstructed routes; it is not an error-free reconstruction of users’ true trajectory-level environmental experiences. A grid-level DBG estimate therefore requires at least one valid reconstructed route. Because origin grids can be supported by different numbers of valid routes, unequal route support is treated as a source of measurement uncertainty.

Land surface temperature (LST) data were obtained from the China Land and Surrounding Areas Daily 1 km All-Weather Land Surface Temperature Dataset (TRIMS LST; 2000–2025), provided by the National Tibetan Plateau Data Center (48). LST observations for May 2025 were extracted and spatially harmonized with the 200 m × 200 m analytical grid. This period was selected to align the thermal-environment data as closely as possible with the Keep trajectory collection window (May 5–18, 2025). Population data were obtained from the WorldPop R2025A v1 population surface for China for 2025, with an approximate native resolution of 100 m; source values were averaged within the 200 m analytical grids and transformed as ln(1 + x) before modelling. Housing-price data were obtained from Lianjia for 2023, harmonized to the 200 m analytical grid, and natural-log transformed before modelling. The traffic variable in the final analytical dataset was derived from the mean value of a Tianjin traffic-facility-service spatial layer intersecting each 200 m grid and is therefore referred to as traffic-facility-service intensity (TFSI), rather than direct traffic congestion. LST and TFSI were included in the environmental-stress domain, while population density and housing price served as socioeconomic and locational controls. Composite indices such as SBG and DBG are used to describe spatial distributions, mismatches, and integrated patterns. To avoid entering the same information twice, the machine-learning models omit the composite indices and use the 12 original or time-weighted predictors retained after prespecified screening (Table 1). NDVI and remote-sensing water proportion contribute to SBG but are not part of the final predictor set. NDVI and remote-sensing water proportion were not entered as independent predictors because the dynamic predictor domain contains the four street-view indicators for which matched time-weighted route counterparts can be constructed. Restricting the static predictor domain to these corresponding indicators preserves the matched static-DBG comparison and avoids adding unmatched remote-sensing predictors to the model.

**Table 1:** Final 12 predictor variables, measurement levels, and model roles.

Dimension	Variable	Measurement level	Model role
Static blue-green	Green View Index (GVI)	Grid	Predictor
Static blue-green	Vegetation-category proportion	Grid	Predictor
Static blue-green	Tree-and-grass visible proportion (TGV)	Grid	Predictor
Static blue-green	Street-view water proportion	Grid	Predictor
DBG	Time-weighted GVI	Reconstructed walking-route process	Predictor
DBG	Time-weighted vegetation-category proportion	Reconstructed walking-route process	Predictor
DBG	Time-weighted TGV	Reconstructed walking-route process	Predictor
DBG	Time-weighted street-view water proportion	Reconstructed walking-route process	Predictor
Environmental stress	Land surface temperature	Grid	Predictor
Environmental stress	Traffic-facility-service intensity (TFSI)	Grid	Predictor
Socioeconomic	Log population density	Grid	Control variable
Socioeconomic	Log housing price	Grid	Control variable

### 2.3 Measurement of walking activity and environmental indicators

Walking activity density measures cumulative Keep trajectory length per unit of effective spatial area (5). After intersecting the trajectories with the regular grid, we calculated and summed the lengths of all trajectory segments within each grid. For grid i , walking activity density (WAD) is defined as:

$${\mathrm{WAD}}_{i}=\frac{L_{i}}{A_{i}}$$

where $L_{i}$ is the cumulative Keep trajectory length within grid i and $A_{i}$ is the grid area used to calculate density. If a grid contains large water bodies, wetlands, or other non-walkable spaces, $A_{i}$ is based on effective land area rather than the original grid area. Because WAD generally has a right-skewed distribution, the machine-learning models use $Y_{i}=\ln(1+{\mathrm{WAD}}_{i})$ as the dependent variable.

Figure 3 traces the construction of WAD. Panel a presents the publicly shared Keep trajectories and preprocessing workflow; panel b maps the cleaned trajectories; panel c shows their intersection with the 200 m × 200 m grid, segmentation, and accumulation of segment lengths; and panel d shows grid-level WAD and its linkage to population-weighted distribution assessment. The figure shows WAD construction only, not the time-weighted calculation of DBG.

**Figure 3:**
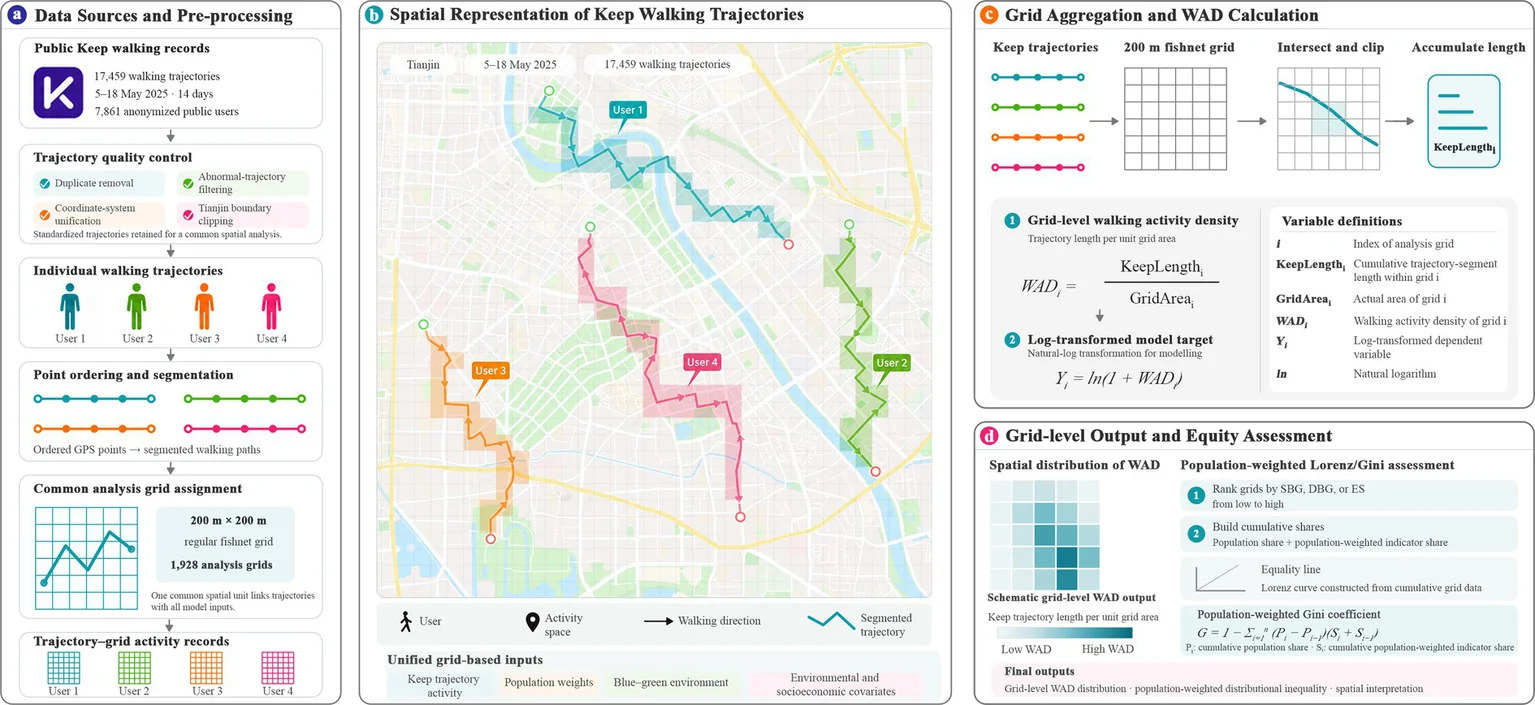
Schematic of Keep walking trajectory preprocessing, spatial representation, grid aggregation, WAD calculation, and population-weighted distribution assessment.

The static blue-green supply index (SBG) describes the blue-green environmental conditions of each grid at the study time point. GVI, NDVI, vegetation-category proportion, TGV, street-view water proportion, and remote-sensing water proportion are treated as positive indicators. We first apply Min-Max normalization to place the measures on a common scale (49):

$$x{'}_{\mathrm{ij}}=\frac{x_{\mathrm{ij}}-\min_{i}x_{\mathrm{ij}}}{\max_{i}x_{\mathrm{ij}}-\min_{i}x_{\mathrm{ij}}}$$

We then calculate entropy weights and the weighted sum:

$${\mathrm{SBG}}_{i}=\sum_{j}w_{j}x{'}_{\mathrm{ij}}$$

Higher SBG values indicate a greater supply of static blue-green resources within the grid. Entropy weights represent differences in the indicators’ information content within the sample, not their theoretical importance. We therefore also construct an equal-weight index for the robustness analysis (49).

The DBG index approximates blue-green conditions along reconstructed walking routes. For environmental indicator j on route r, exposure is weighted by the segment-level walking time returned by the API:

$$E_{\mathrm{rj}}=\frac{\sum_{s}t_{\mathrm{rs}}x_{g(r,s),j}}{\sum_{s}t_{\mathrm{rs}}}$$

$$E_{\mathrm{ij}}=\frac{1}{N_{i}}\sum_{r=1}^{N_{i}}E_{\mathrm{rj}}$$

$${\mathrm{DBG}}_{i}=\sum_{j}v_{j}S(E_{\mathrm{ij}})$$

where $t_{\mathrm{rs}}$ is the API walking time for route r on segment s , $x_{g(r,s),j}$ is the value of the j -th environmental indicator in the grid containing that segment, $N_{i}$ is the number of valid routes within origin grid i , $S(E_{\mathrm{ij}})$ denotes the 0–1 normalized route exposure, and $v_{j}$ is the entropy weight of the j -th indicator. A higher DBG indicates higher estimated overall blue-green exposure along reconstructed walking routes originating from that grid. To assess uncertainty associated with time weighting and route reconstruction, a fixed speed of 5 km·h^−1^ was used to construct length-equivalent weights, which were compared with the API time-weighted results; reconstructed route lengths were also compared with the original Keep polyline lengths. Routes with missing or non-positive distance or time values, or with failed geometry checks, were flagged and excluded.

WAD sums the lengths of trajectories that pass through each grid, whereas DBG is assigned to the route-origin grid. Their spatial supports therefore do not align perfectly at the individual level. Subsequent model results are interpreted only as grid-level spatial associations and relative predictive contributions, not as causal effects of individual exposure on walking behavior.

The environmental stress index (ES) combines land surface temperature and traffic-facility-service intensity (TFSI). TFSI captures traffic-related urban context, while LST represents thermal stress. After Min-Max normalization, we aggregate the two indicators using entropy weights:

$${\mathrm{ES}}_{i}=\sum_{k}u_{k}e{'}_{\mathrm{ik}}$$

Here, $e{'}_{\mathrm{ik}}$ is the normalized value of environmental-stress indicator k in grid i , and $u_{k}$ is its entropy weight. Higher ES values indicate greater environmental stress within the grid.

### 2.4 Spatial distributional inequality and environment-use mismatch assessment

We assess the distributional inequality of SBG, DBG, and ES relative to population separately. For each indicator, grids are ranked from low to high, population weights are derived from population density, and the cumulative population share $P_{i}$ and cumulative population-weighted indicator share $Q_{i}$ are calculated. These values define the population-weighted Lorenz curves and Gini coefficients (14, 21):

$$G=1-\sum_{i=1}^{n}(P_{i}-P_{i-1})(Q_{i}+Q_{i-1})$$

G ranges from 0 to 1. Values near 0 indicate that an indicator is distributed more evenly relative to population, whereas values near 1 indicate greater concentration. For SBG and DBG, a higher G means that higher levels of the blue-green environment are concentrated in spatial units associated with smaller population shares. For ES, a higher G denotes a more uneven distribution of environmental burden but does not imply a lower burden level. After grids with zero population weight were excluded, the analysis included 1,925 grids. To account for potential spatial dependence among neighboring grids, we used 80 spatial blocks of 5 km as resampling units for 1,000 bootstrap resamples with replacement and estimated 95% confidence intervals for the Gini coefficients (50).

We next assess grid-level mismatches between environmental conditions and Keep walking use (17, 19, 20). All variables are normalized to 0–1, with $S(X_{i})$ denoting the standardized value of variable X in grid i . The SBG-use gap, DBG-use gap, and ES-use joint indicator are defined as follows:

$$M_{i}^{s}=S({\mathrm{WAD}}_{i})-S({\mathrm{SBG}}_{i})$$

$$M_{i}^{d}=S({\mathrm{WAD}}_{i})-S({\mathrm{DBG}}_{i})$$

$$M_{i}^{e}=S({\mathrm{WAD}}_{i})\times S({\mathrm{ES}}_{i})$$

When both gaps are positive, Keep walking use is relatively higher than both the corresponding static supply and DBG. A higher stress-use joint indicator denotes stronger co-occurrence of high Keep walking use and high environmental stress.

For a more interpretable descriptive classification, we use medians from the common valid sample to classify the SBG-WAD, DBG-WAD, and ES-WAD relationships into high-high (HH), high-low (HL), low-high (LH), and low-low (LL) quadrants. In the SBG-WAD and DBG-WAD classifications, LH denotes the co-occurrence of relatively low static supply with relatively high Keep walking use and relatively low DBG with relatively high Keep walking use, respectively. In the ES-WAD classification, HH denotes the co-occurrence of relatively high environmental stress with relatively high Keep walking use. These classes are used to identify candidate environment-use mismatch patterns for further examination. “High” and “low” are relative to the study area, and the classification does not replace street-scale field verification.

We also construct a composite Priority Index (PI) to screen jointly for high Keep walking use, low static blue-green supply, low DBG, and high environmental stress:

$${\mathrm{PI}}_{i}=S({\mathrm{WAD}}_{i})\times [1-S({\mathrm{SBG}}_{i})]\times [1-S({\mathrm{DBG}}_{i})]\times S({\mathrm{ES}}_{i})$$

Higher PI values indicate stronger co-occurrence of the four characteristics within a grid. PI is used only for relative spatial screening; it represents neither causal effects nor an objective investment ranking. We divide PI into low-, medium-, and high-priority tertiles to compare the magnitude of differences among priority levels. Kruskal-Wallis $\varepsilon^{2}$ effect sizes summarize group separation in WAD, SBG, DBG, and ES. These comparisons provide descriptive robustness evidence rather than a separate basis for causal inference. Because its multiplicative form, normalization, and weighting choices are analytical decisions, PI is interpreted as a relative screening tool whose rankings may vary under alternative specifications, rather than as an objective or transferable planning-priority standard.

### 2.5 Data quality, correlation, and multicollinearity

For each model variable, we calculate missingness and flag potential outliers using the 1.5 × IQR rule and ∣z∣>3 . A single statistical threshold does not trigger automatic removal; instead, histograms, density plots, and standardized boxplots are used to examine distribution and plausibility. We then calculate Pearson and Spearman correlations among the 12 predictors and compute variance inflation factors (VIFs) from a linear model with an intercept. VIF > 10 indicates high multicollinearity. The correlation matrix and VIFs also inform interpretation of subsequent SHAP and joint-permutation results, so information shared among correlated variables is not treated as an independent effect of any one predictor. The highly correlated visual-green predictors are retained because the modeling objective is prediction and domain-level comparison rather than independent coefficient estimation. High collinearity can redistribute SHAP attribution among correlated predictors. We therefore interpret variable-level rankings cautiously and emphasize domain-level SHAP aggregation and joint-domain permutation.

### 2.6 Model comparison, interpretation, and robustness analysis

We compare Linear Regression, Random Forest, XGBoost, and LightGBM using the same samples and data splits (33, 35). The primary validation is five-fold GroupKFold based on 5 km spatial blocks, which reduces spatial leakage between the training and test sets; random five-fold KFold provides a comparison (50). Performance is measured by $R^{2}$ , RMSE, and MAE, with mean spatial five-fold $R^{2}$ as the primary model-selection criterion. Because only five matched folds are available and the focus is out-of-area predictive behavior, between-model comparisons rely on fold-level performance patterns rather than formal significance tests.

Random Forest uses 600 trees, a minimum leaf size of 4, and a feature-subsampling ratio of 0.8. XGBoost and LightGBM each use 650 boosting rounds and a learning rate of 0.025. Within each spatial fold, the selected model is refitted on the training data and interpreted only for the held-out grids to produce out-of-fold explanations. Interpretation includes TreeSHAP, out-of-fold SHAP dependence plots, domain-level SHAP aggregation, joint permutation of predictor domains, and TreeSHAP interaction analysis (34, 51, 52). LOWESS trends in the SHAP dependence plots include 95% spatial-block bootstrap intervals based on the same 5 km block structure. The correlation matrix and VIFs are used to assess information shared among correlated variables, and all results are interpreted as predictive associations rather than causal effects.

Given the limited spatial predictive performance of the selected model, SHAP dependence curves are used to identify broad nonlinear patterns and apparent turning points rather than precise or transferable planning thresholds.

We test spatial autocorrelation in out-of-fold residuals with an 8-nearest-neighbor weight matrix based on grid centroids and assess global Moran’s I using 999 permutations. Robustness analyses include 1,000 bootstrap resamples based on 80 spatial blocks of 5 km to estimate uncertainty in model performance and Gini coefficients; comparisons of PI under alternative normalization and weighting schemes; comparisons of PI tertiles using $\varepsilon^{2}$ effect sizes; and a route audit that compares API time weighting with fixed-speed weighting and reconstructed route lengths with original Keep trajectory lengths.

## 3 Results

### 3.1 Sample characteristics and spatial patterns

The final analysis comprised 1,928 common valid 200 m × 200 m grids, grouped into 80 spatial blocks of 5 km. The full model included 12 predictors and no missing values. The mean of $\ln(1+{\mathrm{WAD}}_{i})$ was 11.687 (SD = 1.487), close to the median of 11.781, showing that the log transformation substantially reduced the right skew in walking activity density. By contrast, SBG, DBG, ES, and PI all had means above their medians: 0.029 versus 0.019 for SBG, 0.078 versus 0.058 for DBG, 0.189 versus 0.131 for ES, and 0.104 versus 0.068 for PI (Table 2). High values of static blue-green supply, DBG, environmental stress, and composite priority were therefore concentrated in a subset of grids. Using medians to define the mismatch quadrants can limit the influence of a small number of extreme values on classification.

**Table 2:** Descriptive Statistics of the Main Analytical Variables.

Variable	*n*	Mean	SD	Median	IQR
$\ln(1+{\mathrm{WAD}}_{i})$	1,928	11.687	1.487	11.781	1.764
SBG	1,928	0.029	0.040	0.019	0.024
DBG	1,928	0.078	0.065	0.058	0.054
ES	1,928	0.189	0.158	0.131	0.172
PI	1,928	0.104	0.099	0.068	0.101

The three composite environmental indicators followed distinct patch-like spatial patterns, with only partial overlap among high-value grids (Figure 4). High values of static blue-green supply were relatively dispersed (Figure 4A), consistent with local variation in grid-level blue-green resources. High values of DBG were more scattered (Figure 4B), showing that exposure along routes from different origins did not simply mirror the static environment of each origin grid. By contrast, environmental stress formed more continuous high-value clusters in the main areas of urban activity (Figure 4C). The maps also revealed mismatches: some grids with high static supply did not have equally high DBG, and some areas with high DBG also had high environmental stress. SBG, DBG, and ES therefore capture different dimensions and are examined separately in the subsequent equity and environment-use mismatch analyses.

**Figure 4:**
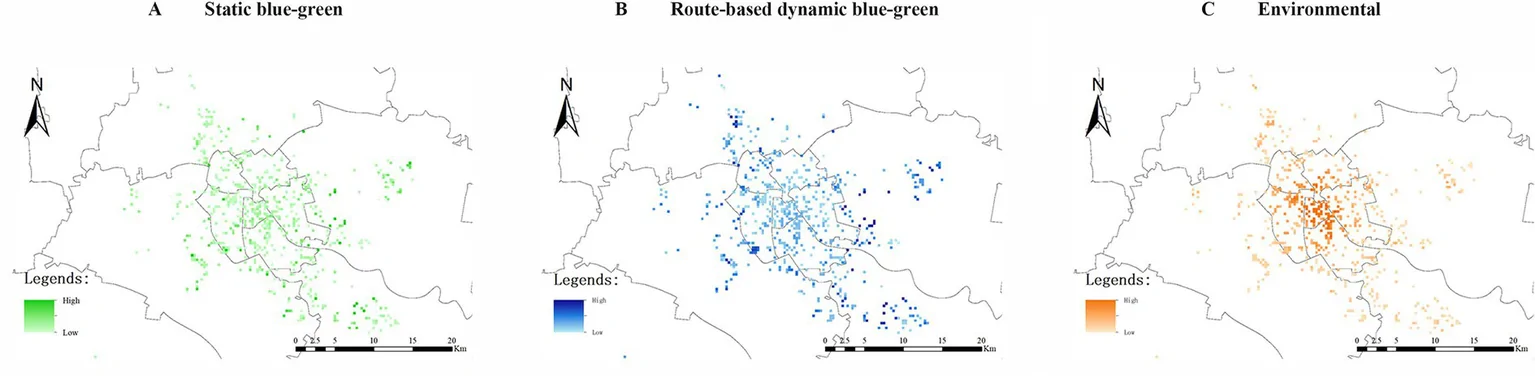
Spatial distribution of composite environmental indicators across 1,928 common valid grids. **(A)**, static blue-green supply (SBG); **(B)**, DBG; **(C)**, environmental stress (ES). Darker colors indicate higher values of the corresponding indicator.

The Spearman correlations were 0.962 between static GVI and static TGV and 0.978 between dynamic GVI and dynamic TGV. The corresponding VIFs ranged from 23.63 to 31.75, whereas those of all other variables did not exceed 2.15. These values indicate severe collinearity among the paired visual-green predictors, while correlations among socioeconomic and environmental stress variables were only moderate. Because shared information can be distributed across correlated variables in SHAP, individual rankings are interpreted cautiously and domain-level results are emphasized in the following analyses.

### 3.2 Spatial distribution of static supply, DBG, and environmental stress

After three grids with zero population weight were excluded, the population-weighted analysis covered 1,925 grids. SBG had the highest Gini coefficient at 0.448 (95% CI: 0.409 to 0.497), followed by ES at 0.374 (0.302 to 0.410) and DBG at 0.331 (0.284 to 0.373), the lowest value (Table 3). The point-estimate differences were 0.117 between SBG and DBG, 0.074 between SBG and ES, and 0.043 between ES and DBG. Thus, static blue-green supply was the most concentrated relative to the population distribution, whereas DBG was more evenly distributed. Because the three confidence intervals overlap to varying degrees, these results describe a ranking of distributional inequality rather than untested significant differences.

**Table 3:** Population-weighted Gini coefficients of the three composite environmental indicators.

Indicator	Gini coefficient	95% block-bootstrap CI
SBG	0.448	0.409 to 0.497
DBG	0.331	0.284 to 0.373
ES	0.374	0.302 to 0.410

The Lorenz curves followed the same relative pattern as the Gini coefficients (Figure 5). Across most of the cumulative population range, the SBG curve lay farthest from the line of perfect equality, showing that static blue-green resources were concentrated in grids representing smaller population shares. The DBG curve lay closest to the equality line overall, consistent with a comparatively more even population-based distribution of DBG estimated from reconstructed walking routes. The ES curve generally fell between the other two. Thus, the amount of blue-green resource within a grid and DBG estimated from reconstructed walking routes originating from that grid have different spatial distributions. This distinction supports the separate description of low-SBG/high-use and low-DBG/high-use spatial co-occurrence patterns in subsequent analyses. The lower Gini coefficient of ES should therefore be read only as greater distributional evenness, not as evidence of a lower environmental burden or greater environmental equity.

**Figure 5:**
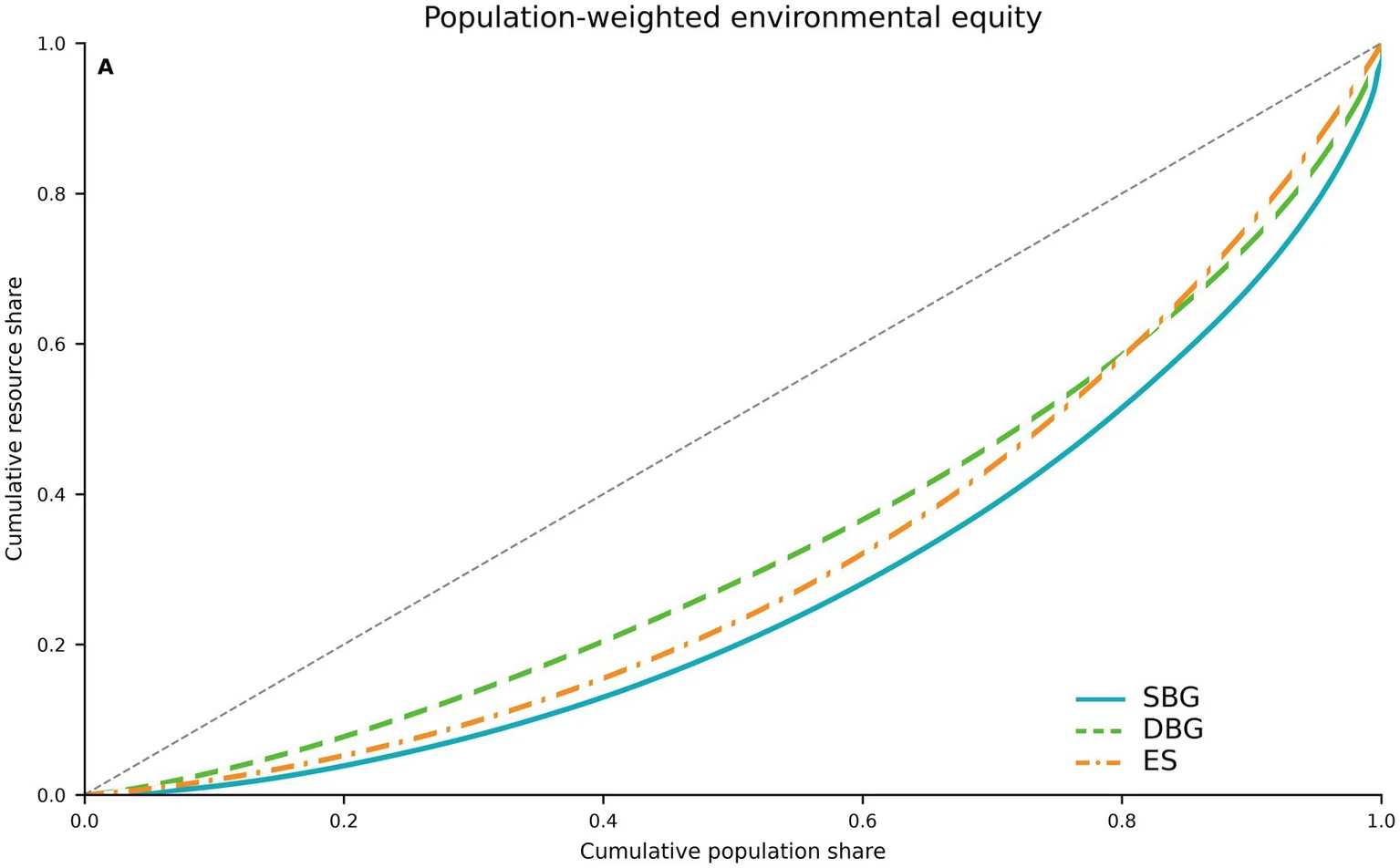
Population-weighted Lorenz curves for static blue-green supply, DBG, and environmental stress. The gray dashed line represents perfect equality; SBG is shown with a solid line, DBG with a dashed line, and ES with a dash-dot line.

### 3.3 Environment-walking use mismatch and priority areas

Each environmental indicator and raw WAD were divided at their medians to form four quadrants, with all three classifications covering the full set of 1,928 grids. In the SBG-WAD classification, 448 grids (23.2%) had low SBG and high WAD. The corresponding DBG-WAD class contained 492 grids (25.5%), 44 more than the SBG-WAD class, and the high ES-high WAD class contained 530 grids (27.5%; Table 4). These classes describe the co-occurrence of above-median platform-recorded walking use with below-median SBG, below-median DBG, or above-median ES, respectively. Although the counts were similar, they were not identical, so a single environmental dimension would not capture all candidate mismatch patterns. Because WAD accumulates trajectories across traversed grids whereas DBG is summarized by route-origin grid, the DBG-WAD class represents a grid-level mismatch between differently supported measures rather than an individual-level exposure-behavior pairing.

**Table 4:** Three priority mismatch classes and their overlap with the top 10% of PI.

Relationship	Priority mismatch class	*n*	Percentage	Overlap with top 10% of PI, *n* (%)
SBG-WAD	Low SBG-high WAD	448	23.2%	90 (46.6%)
DBG-WAD	Low DBG-high WAD	492	25.5%	123 (63.7%)
ES-WAD	High ES-high WAD	530	27.5%	152 (78.8%)

Percentages in the final column use the 193 grids in the top 10% of PI as the denominator.

The three candidate mismatch classes are spatially interwoven, but they do not form identical continuous clusters (Figures 6A–C). Some grids fall into both the low SBG/high WAD and low DBG/high WAD classes, whereas each class also includes distinct grids. The two classifications therefore identify partly different spatial co-occurrence patterns. High ES/high WAD grids are more widely distributed across high-activity areas. The continuous PI combines high WAD, low SBG, low DBG, and high ES (Figure 6D). High PI values cluster in the major high-activity areas, with scattered patches in peripheral areas. The top 10% comprises 193 grids: 152 (78.8%) also have high ES/high WAD, 123 (63.7%) have low DBG/high WAD, and 90 (46.6%) have low SBG/high WAD (Table 4). High-PI grids thus frequently combine several adverse or potentially adverse spatial characteristics, most commonly high ES and high WAD.

**Figure 6:**
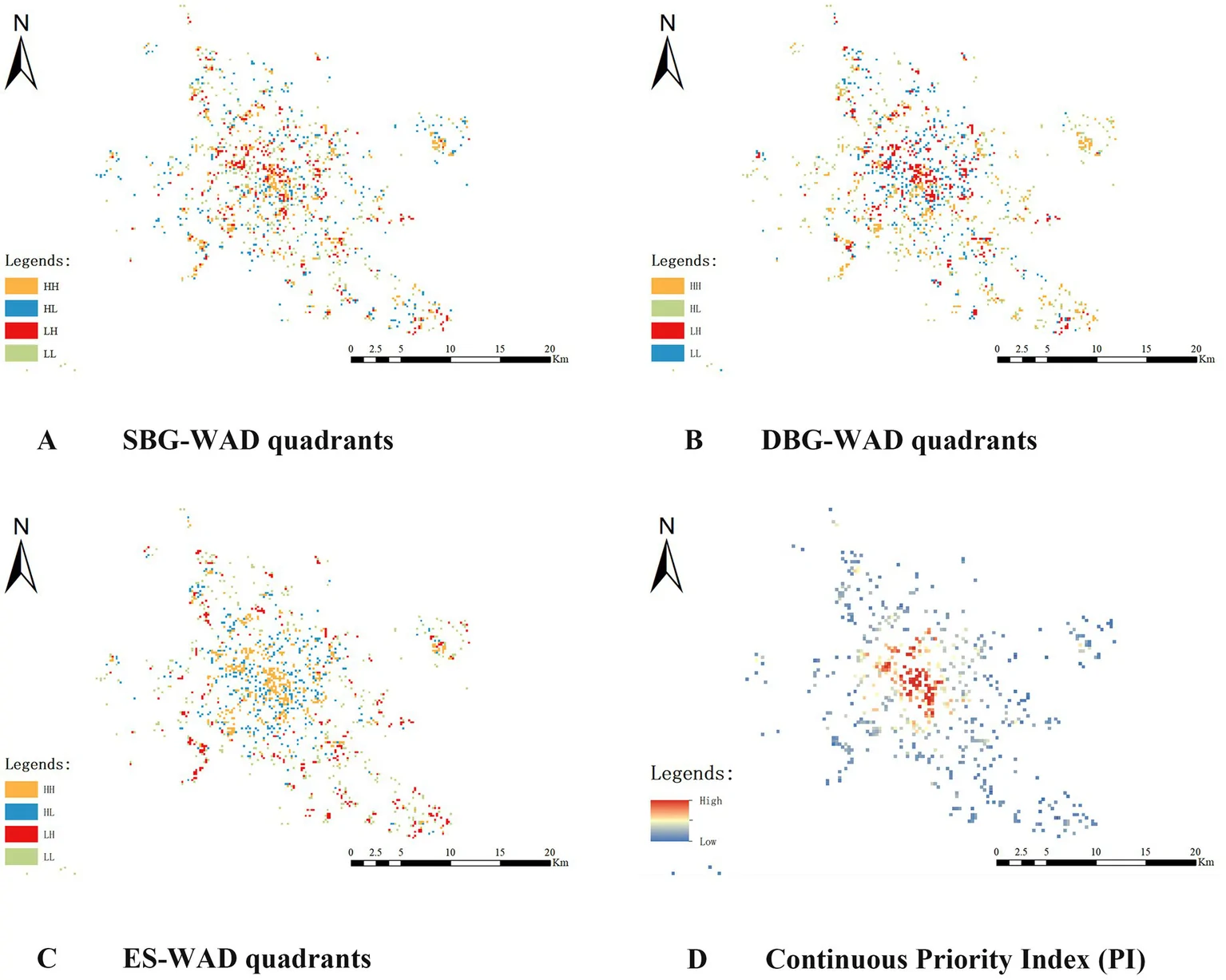
Mismatches between environmental conditions and walking activity density, with the continuous priority index. **(A)**, SBG-WAD quadrants; **(B)**, DBG-WAD quadrants; **(C)**, ES-WAD quadrants; **(D)**, continuous priority index (PI). Black outlines mark key mismatch classes and grids in the top 10% of PI.

The quadrant classifications and PI are complementary. The quadrants identify the primary mismatch type, whereas the continuous PI compares overall priority levels across grids. Together, they help screen a large set of candidate grids for areas requiring priority field verification while retaining information about the type of problem.

### 3.4 Model performance under random and spatial cross-validation

All four algorithms were evaluated using the same samples and folds (Table 5). Under random five-fold cross-validation, Random Forest had the highest $R^{2}$ (0.114 ± 0.034), followed by XGBoost (0.089 ± 0.049), LightGBM (0.084 ± 0.056), and Linear Regression (0.026 ± 0.033). Their corresponding RMSE values were 1.396, 1.415, 1.419, and 1.464. Under spatial five-fold cross-validation, $R^{2}$ declined markedly for all three tree-based models: Random Forest fell to 0.051 ± 0.027, XGBoost to 0.024 ± 0.016, and LightGBM to −0.003 ± 0.032. Linear Regression remained close to its random-split result, with an $R^{2}$ of 0.029 ± 0.025. Random Forest also had the lowest spatial RMSE (1.446), and its MAE of 1.118 was comparable to that of Linear Regression. Given its combined $R^{2}$ and RMSE performance, Random Forest was selected for subsequent interpretation.

**Table 5:** Performance of the four algorithms under random and spatial five-fold cross-validation.

Model	Validation scheme	*R^2^*	RMSE	MAE
Random forest	Random five-fold	0.114 ± 0.034	1.396	1.065
XGBoost	Random five-fold	0.089 ± 0.049	1.415	1.079
LightGBM	Random five-fold	0.084 ± 0.056	1.419	1.080
Linear regression	Random five-fold	0.026 ± 0.033	1.464	1.117
Random forest	Spatial five-fold	0.051 ± 0.027	1.446	1.118
Linear regression	Spatial five-fold	0.029 ± 0.025	1.463	1.118
XGBoost	Spatial five-fold	0.024 ± 0.016	1.466	1.139
LightGBM	Spatial five-fold	−0.003 ± 0.032	1.487	1.158

The contrast between validation strategies is evident in Figure 7. Random Forest, XGBoost, and LightGBM all had higher $R^{2}$ values and lower RMSE values under random five-fold cross-validation than under spatial five-fold cross-validation. For Random Forest, $R^{2}$ fell from 0.114 to 0.051. Linear Regression changed little in either metric. These results suggest that random splits allow tree-based models to exploit more local spatial similarity, but their ability to generalize to new areas weakens when entire 5-km spatial blocks are held out from model fitting. However, the absolute spatial R^2^ remained low, so subsequent model interpretation is treated as exploratory and is not used to derive stable planning thresholds.

**Figure 7:**
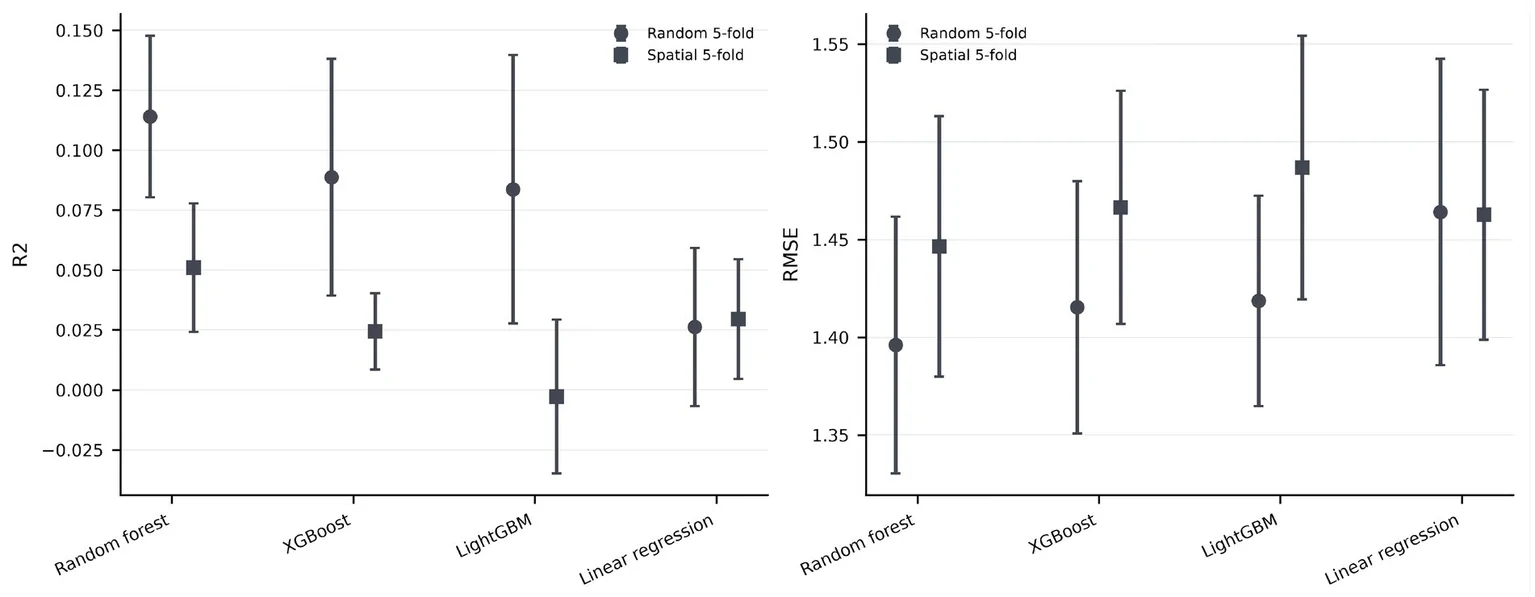
$R^{2}$ and RMSE of the four algorithms under random and spatial five-fold cross-validation. Error bars indicate the standard deviation across the five folds.

### 3.5 Predictive contributions and nonlinear associations

In the out-of-fold SHAP analysis for Random Forest, TFSI had the largest mean absolute predictive contribution (0.167), followed by static TGV (0.144), log population density (0.133), log housing price (0.130), and dynamic vegetation-category proportion (0.125). Land surface temperature ranked sixth. Dynamic TGV, dynamic GVI, and static GVI had intermediate predictive contributions, whereas static vegetation-category proportion and the two street-view water indicators had relatively small predictive contributions overall. This variable-level ranking shows that the model drew simultaneously on environmental stress, static blue-green supply, socioeconomic factors, and DBG. Because some visual-green variables were highly correlated, individual-variable rankings are considered alongside the domain-level results. Given the spatial five-fold R^2^ of only 0.051 ± 0.027, these rankings are used as relative predictive summaries within a model with limited spatial generalizability.

When aggregated by domain, socioeconomic factors accounted for 27.2% of the SHAP predictive contribution, DBG for 25.0%, environmental stress for 24.2%, and static blue-green supply for 23.7%, a maximum difference of only 3.5 percentage points (Figure 8A). Joint permutation measured the loss in model performance when each domain was disrupted. Environmental stress produced the largest decrease in spatial-test-fold $R^{2}$ (0.058), followed by static blue-green supply (0.046), socioeconomic factors (0.045), and DBG (0.042; Figure 8B). The two methods yielded different rankings because SHAP allocates predictive contributions within the fitted model, whereas joint permutation measures performance loss after disrupting a domain. Such differences are expected when variables are correlated and domains contain overlapping information. Both methods nevertheless show that all four domains provide predictive information and that no single domain is overwhelmingly dominant.

**Figure 8:**
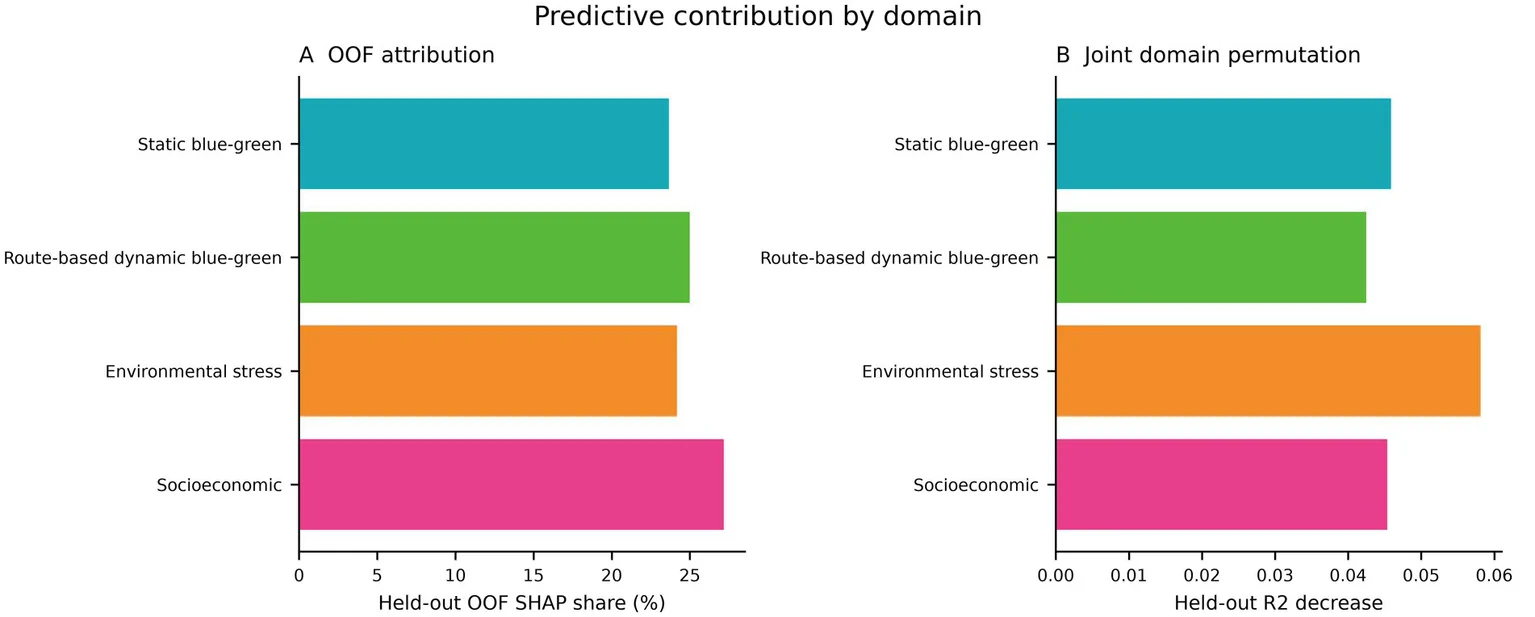
Predictive contributions of the four variable domains. **(A)**, out-of-fold SHAP contribution shares; **(B)**, decrease in $R^{2}$ after jointly permuting one domain within the spatial test folds.

The out-of-fold SHAP dependence plots indicated broad nonlinear and non-monotonic patterns, including apparent turning points, plateaus, and changes in direction (Figure 9). Given the limited spatial cross-validation performance of Random Forest, these curves are used to describe qualitative predictive patterns rather than precise, stable, or transferable thresholds.

**Figure 9:**
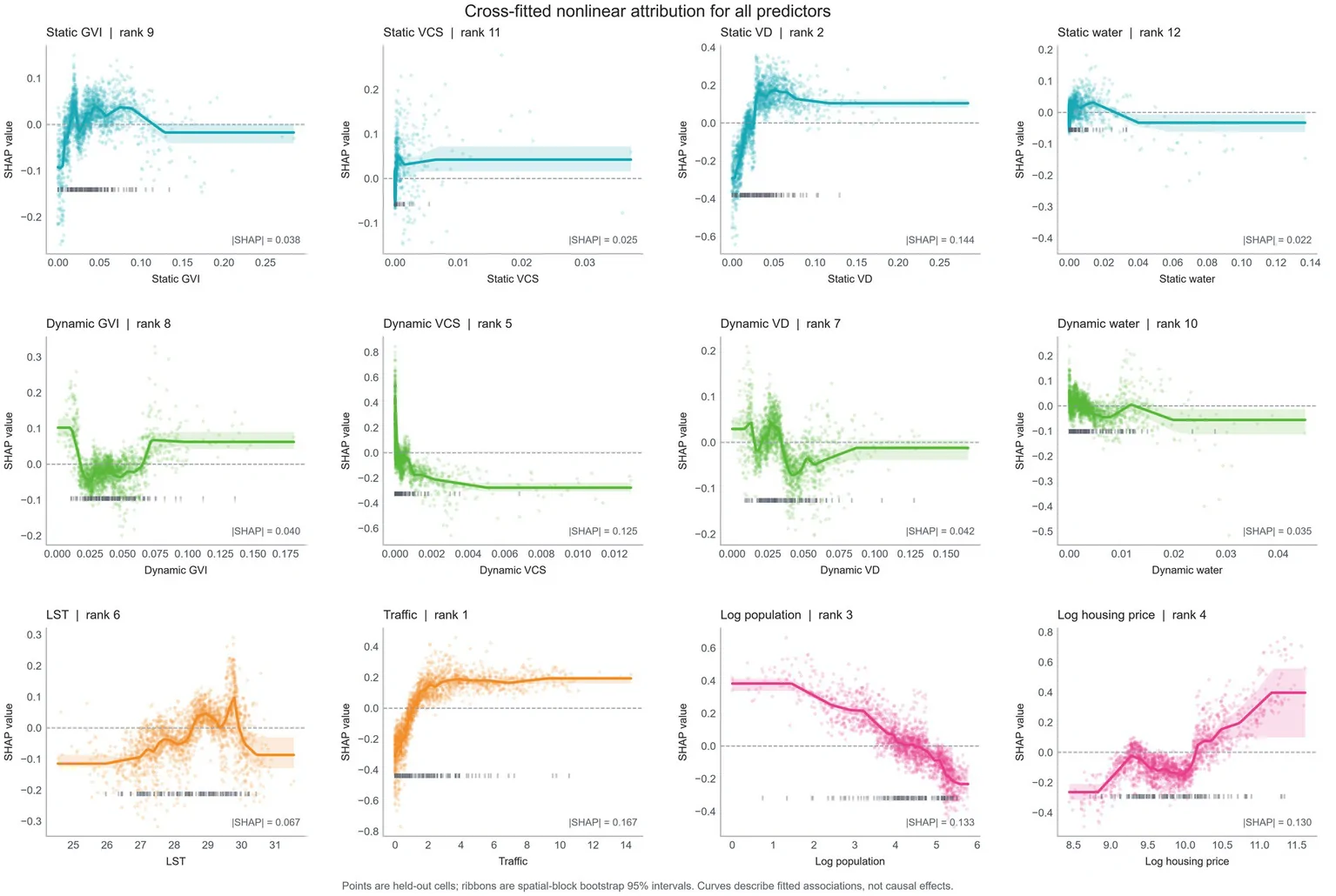
Out-of-fold SHAP dependence plots for the 12 predictors. From left to right and top to bottom: static GVI, static vegetation-category proportion, static TGV, static water proportion, dynamic GVI, dynamic vegetation-category proportion, dynamic TGV, dynamic water proportion, land surface temperature, TFSI, log population density, and log housing price. Points represent spatially held-out grids, the solid line is the LOWESS trend, and the shaded band is the 95% spatial-block bootstrap interval; the mean absolute SHAP value and importance rank are shown in the lower-right corner of each panel.

Among the static blue-green predictors, static TGV had the largest mean absolute SHAP value (0.144, rank 2), whereas static GVI, vegetation-category proportion, and street-view water proportion had smaller predictive contributions. The dependence curves were non-monotonic: GVI changed direction across its observed range, vegetation-category proportion quickly approached a plateau, TGV rose strongly from low values before its predictive contribution weakened, and water proportion showed only small changes around zero. These shapes are interpreted as broad predictive patterns rather than stable cutoff values or causal effects.

Among the DBG predictors, dynamic vegetation-category proportion had the largest predictive contribution (|SHAP| = 0.125, rank 5), followed by dynamic TGV (0.042), dynamic GVI (0.040), and dynamic water proportion (0.035). Their dependence curves were also non-monotonic: dynamic GVI showed a decline-then-rise pattern, dynamic vegetation-category proportion declined rapidly at the low end and then flattened, dynamic TGV showed a trough followed by partial recovery, and dynamic water proportion remained comparatively weak. These patterns indicate heterogeneous predictive associations rather than fixed exposure thresholds or causal effects.

Among the environmental-stress predictors, land surface temperature (|SHAP| = 0.067, rank 6) showed a local inverted-U predictive pattern, whereas TFSI had the largest predictive contribution overall (|SHAP| = 0.167, rank 1), although its SHAP dependence varied nonlinearly across the observed range. The TFSI pattern likely reflects broader urban context, including activity intensity, centrality, destination clustering, facility concentration, and other unmeasured spatial characteristics.

The socioeconomic variables also had substantial predictive contributions. Log population density (|SHAP| = 0.133, rank 3) and log housing price (|SHAP| = 0.130, rank 4) showed heterogeneous SHAP patterns across their observed ranges. These patterns may reflect multiple correlated aspects of urban centrality, facility provision, and population context rather than stable thresholds or independent causal effects.

Across the 12 panels, no predictor showed a simple and consistently monotonic association with model output. The curves instead suggested broad nonlinear patterns, including apparent turning points, plateaus, changes in direction, and local peaks. Because spatial predictive performance was limited and observations were sparse at the tails of some distributions, these patterns should be treated as exploratory model features rather than precise planning thresholds. They describe predictive associations in spatially held-out grids, not causal effects of environmental variables on walking behavior.

Of the 66 variable pairs, log population density and log housing price had the strongest interaction, with a mean absolute interaction value of 0.031 and a fold-wise rank stability of 0.94 (Figure 10). The next strongest interactions were static TGV × population density (approximately 0.023), land surface temperature × housing price, and TFSI × population density (both approximately 0.022). Most of the 10 strongest interactions linked socioeconomic, environmental stress, and static blue-green supply variables, indicating predictive interactions among these factors. The conditional dependence plots show that the downward trend for population density persists across housing-price levels. They also show that static TGV rises rapidly in the low-value range and that its SHAP contribution distribution varies across population-density levels.

**Figure 10:**
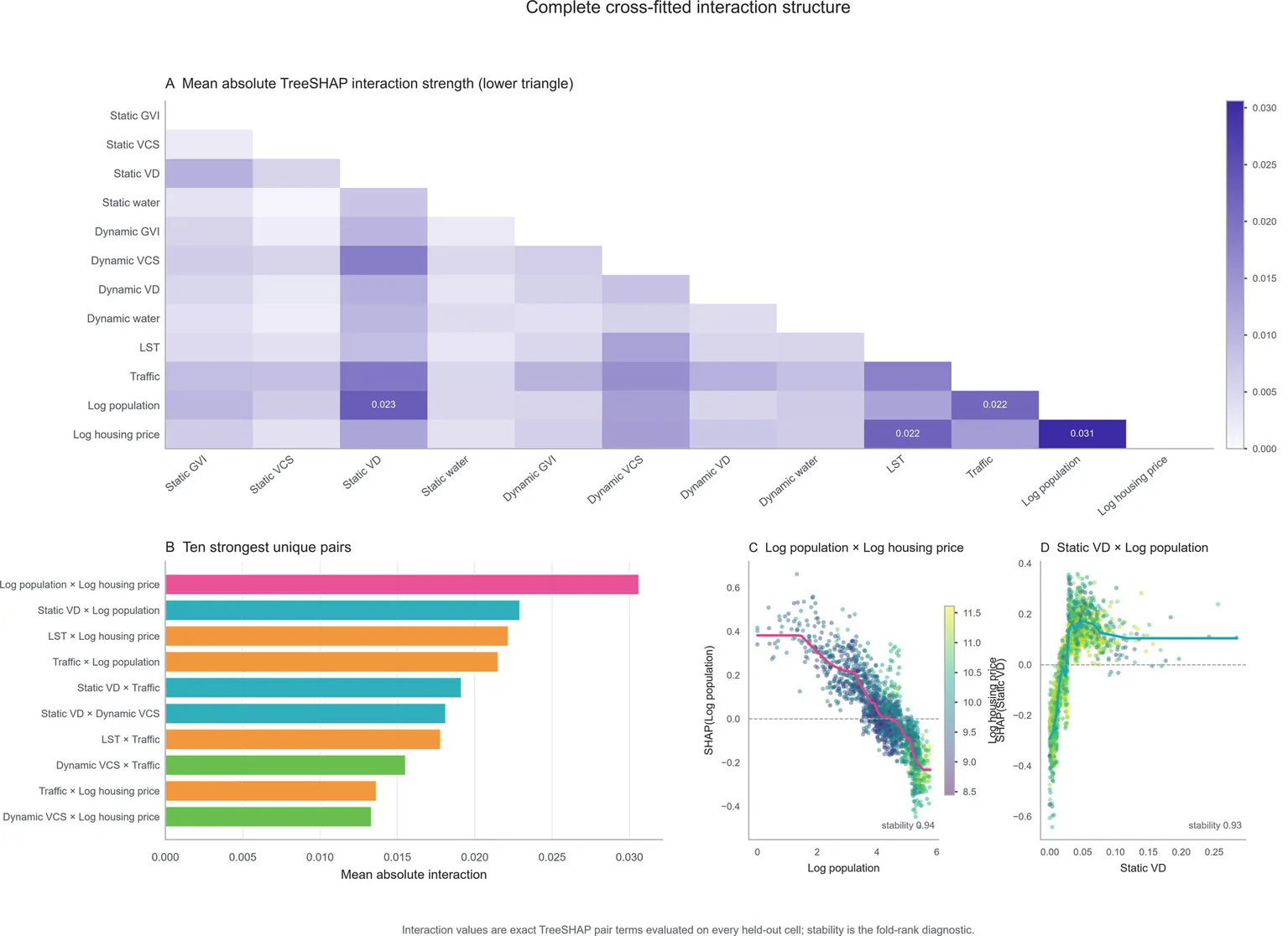
Out-of-fold TreeSHAP interaction structure among the 12 variables. **(A)**, matrix of mean absolute interaction strength for all 66 variable pairs; **(B)**, the 10 strongest interactions; **(C,D)**, two top-ranked and fold-stable conditional dependence relationships.

### 3.6 Residual spatial structure and robustness

The out-of-fold residuals from Random Forest under spatial five-fold cross-validation retained positive spatial autocorrelation (Moran’s I = 0.166, p = 0.001). In Figure 11A, positive and negative residuals formed same-sign clusters in several neighboring areas rather than a random spatial pattern. The positive slope of the Moran scatterplot in Figure 11B likewise shows that high residuals tended to be adjacent to high residuals, while low residuals tended to be adjacent to low residuals.

**Figure 11:**
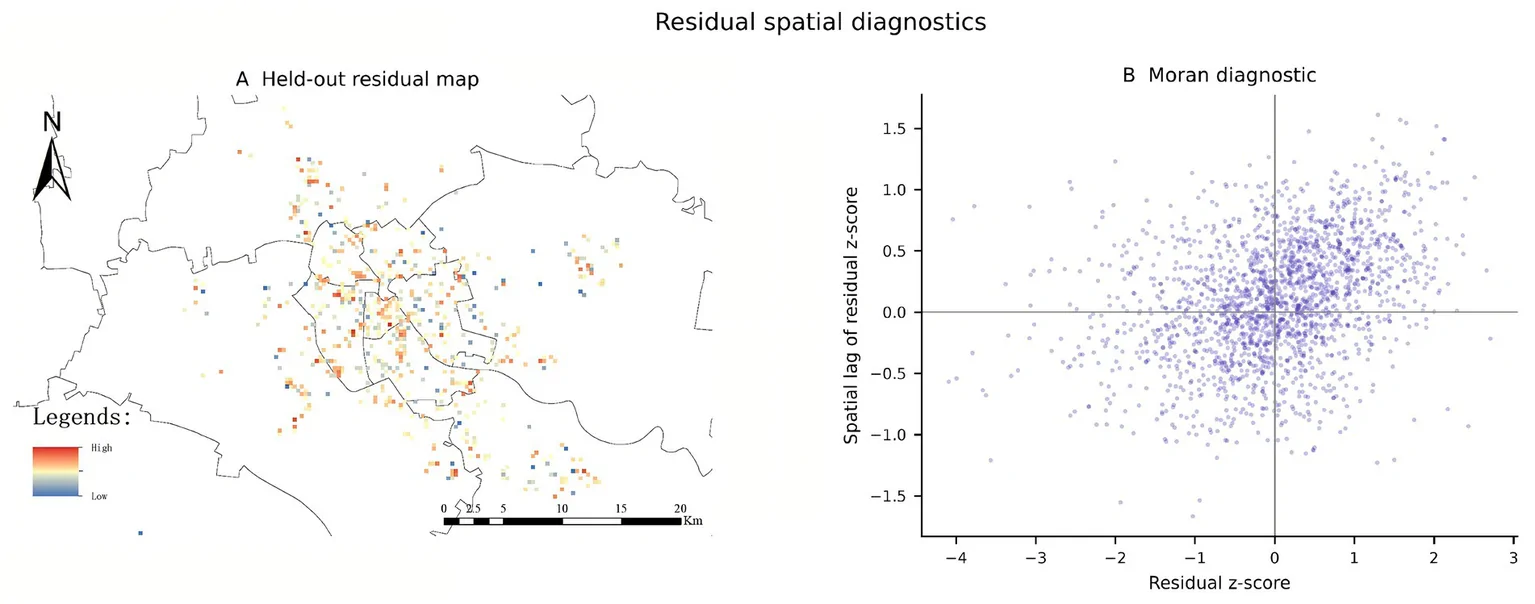
Out-of-fold residual diagnostics for spatial five-fold cross-validation. **(A)**, spatial distribution of standardized residuals; **(B)**, Moran scatterplot of residuals.

Spatial-block bootstrap analysis yielded a Random Forest $R^{2}$ of 0.051 (95% CI, 0.014 to 0.081), consistent with the main point estimate. The entire interval remained above 0. Although the full model had limited spatial predictive performance, the direction of its improvement over the mean baseline was broadly stable under block resampling. Bootstrap estimates for the three Gini coefficients also preserved the ranking of SBG as highest and DBG as lowest. For the priority index, the Spearman correlation between the 1 to 99% robust-normalization scheme and the baseline PI was 0.975, so the treatment of extreme values had little effect on the overall ranking. Under equal weighting, the correlation fell to 0.808, showing that the weighting scheme had a more noticeable effect on some top-ranked grids. In the tertile comparison of PI groups, ES had the largest effect size ( $\varepsilon^{2}$ = 0.815), followed by DBG (0.128) and WAD (0.101), while SBG was smaller (0.024). High PI values were therefore distinguished primarily by high ES, high WAD, and lower DBG. This result supports using PI as a screening indicator rather than as an objective investment ranking.

The route audit covered 4,725 assigned reconstructed routes, of which 4,724 passed the geometry check. These routes represent only the reconstructed-route subset, not the full set of publicly shared Keep trajectory records. With reconstructed geometry held constant, all Spearman correlations between fixed-speed and API time-weighted exposure exceeded 0.9999. The time-weighting specification therefore had almost no effect on the relative ranking of DBG. By contrast, the median ratio of reconstructed route length to original Keep trajectory length was 3.667 (IQR: 2.932 to 4.736). Route-geometry reconstruction thus introduced substantially more measurement uncertainty than the time-weighting specification itself.

## 4 Discussion

### 4.1 Main findings

The main methodological contribution of this study is to distinguish four constructs often conflated in spatial assessments. These are fixed-grid blue-green supply, reconstructed-route DBG, environmental stress, and platform-recorded walking use. The framework compares how these dimensions align or diverge, while the machine-learning analysis provides supplementary predictive evidence under spatial validation. Fixed-grid resource supply should not be treated as equivalent to environmental conditions encountered during movement or to observed use (14, 20).

The four measures answer different descriptive questions. Static supply represents the relatively stable resource base within a grid. DBG approximates conditions along reconstructed movement paths (22, 23). WAD represents the spatial intensity of use recorded in publicly shared platform data (5, 27, 40, 53), and ES operationally combines thermal stress with traffic-related urban context in areas where walking was recorded (6, 54). The framework therefore examines their spatial alignment, divergence, and predictive association across the four dimensions.

### 4.2 Why static supply and DBG differ

SBG and DBG differ because they measure different spatial contexts. SBG describes the blue-green environment within a grid and therefore has relatively fixed spatial support (22, 25). DBG estimates conditions along reconstructed walking routes originating in that grid, so it depends on cross-grid movement, route length, route choice, and the reconstruction method. This distinction implies that environmental conditions relevant to walking are scale- and mobility-dependent: fixed-grid resource availability and movement-mediated exposure are analytically distinct constructs rather than interchangeable proxies. Activity-space research has shown that residential locations and fixed buffers may miss environments encountered during movement. Route-scale measures can complement these place-based measures with estimates of exposure along the movement process (23, 26, 37).

DBG was distributed more evenly relative to the population than SBG, which may reflect both statistical aggregation and behavioral selection. Routes cross several grids, and averaging routes from the same origin reduces local extremes. Walkers may also select routes that are more comfortable, continuous, or visually attractive, so route exposure may partly reflect behavioral choice (22, 26). The lower inequality of DBG means only that reconstructed-route DBG was more evenly distributed within this platform-route sample; it does not show that the dynamic environment was more equitable in a normative sense. Nor is DBG exogenous to walking choice. Its predictive contribution is best interpreted as spatial covariation between the route environment and platform-recorded walking use, not as an independent effect on walking behavior (55).

The mismatch classes in Figure 6 further illustrate this measurement distinction. Low SBG-high WAD grids combine relatively high use with relatively low static resource values, whereas low DBG-high WAD grids combine relatively high use with lower reconstructed-route blue-green exposure (14, 20). Because the two classes only partly overlap, they identify different spatial configurations. These configurations may reflect resource location, pedestrian-network configuration, cross-grid movement, route choice, and measurement support. Areas with low static supply but higher DBG, for example, may reflect cross-grid movement or route selection. Assessments should therefore measure fixed-grid resource availability and movement-based exposure separately (19, 20).

### 4.3 Nonlinear relationships and joint effects

These machine-learning results are complementary to the spatial mismatch framework and should be interpreted conservatively given the limited spatial cross-validation performance of Random Forest. Domain-level SHAP and joint-permutation results showed that static blue-green, DBG, environmental stress, and socioeconomic information each contributed predictive information, with no single domain dominating. Spatial variation in Keep walking use was therefore associated with a combination of urban function, environmental quality, reconstructed-route exposure, and population context (2, 9, 28, 30). It cannot be reduced to a simple claim that more greenery produces more walking or that one environmental stressor determines walking (29, 31, 56).

Figure 9 shows that most variables were not linearly related to model output. TFSI had the highest predictive importance, and its predictive contribution rose sharply from very low values before approaching a plateau. TFSI may partly capture broader spatial characteristics such as urban activity intensity, centrality, destination clustering, and road-network intensity, which may contribute to its strong predictive role (2, 5).

The blue-green curves indicate that model response varied with environmental form and continuity rather than with quantity alone (46). The predictive contribution of static TGV rose quickly from low to moderate levels and then plateaued, while static and dynamic GVI followed different non-monotonic patterns. Average visible greenery within a grid is therefore not equivalent to greenery encountered continuously along a reconstructed route (30, 41, 43). The associations between greenery and walking use may also depend on street function, route choice, and combinations of environmental conditions (32, 45, 56).

The segmented patterns for population density, housing price, and land surface temperature place platform-recorded walking use within broader urban and temporal contexts (2, 9). Population density did not remain positively associated with model output as values increased. Housing price also showed segmented changes that may reflect a combination of density, centrality, facility provision, and housing-market conditions. Land surface temperature rose through the middle range and declined at higher values, a pattern that may relate to the seasonal and thermal conditions of the May sample (54).

### 4.4 Model limits under spatial generalization

The contrast between random and spatial five-fold cross-validation in Figure 7 defines an important limit of the model. Random splitting can place adjacent grids in both the training and test sets, allowing the model to exploit similarities in local roads, urban functions, and population structure and potentially overstate performance. Spatial five-fold cross-validation holds out entire geographic areas and tests whether the model generalizes to locations absent from training (50). Under this stricter scheme, Random Forest achieved $R^{2}$ = 0.051 ± 0.027. The predictors therefore carried some information across areas, but overall spatial generalizability remained limited (55).

Positive spatial autocorrelation remained in the out-of-fold residuals (Figure 11). The model may therefore have omitted spatially structured urban functions and behavioral contexts rather than only random noise (50). Unmeasured factors may include destination mix, street-network connectivity, public transport, crossing facilities, air pollution, noise, weather, time of day, and walking purpose (2, 6, 9). The age, exercise habits, and recording preferences of platform users may also shape the spatial distribution of recorded activity (27).

### 4.5 Using spatial mismatch to screen candidate planning areas

Figure 6 distinguishes the location of a mismatch from its type (19, 20). These mismatch classes are better used to determine what should be verified locally than to prescribe interventions directly. For low SBG-high WAD grids, field audits can examine existing tree canopy, small green spaces, waterfront access, and the accessibility of nearby blue-green resources (15–17, 57). For low DBG-high WAD grids, route-level audits can examine shade continuity, pedestrian-network detours, barriers, and connections between blue-green nodes and frequently used routes (7, 41, 42). For high ES-high WAD grids, local verification can focus on thermal conditions, traffic-facility-service context, and crossing environments. The three classes are interwoven but only partly overlap, so no single greening measure can be inferred from the mismatch maps alone.

The continuous PI screens candidate areas where high use coincides with low static supply, low DBG, and high environmental stress. High-PI grids overlapped most strongly with the high ES-high WAD class, so the index mainly identified locations with substantial recorded walking use and elevated ES values. Its multiplicative form, normalization method, weighting scheme, and candidate threshold are analytical choices, and the equal-weight sensitivity analysis changed some rankings (Spearman correlation with the baseline PI = 0.808). PI should therefore be interpreted only as a relative screening tool, not as an objective or transferable planning-priority standard. In Tianjin, the central high-activity clusters identified in Figure 6 can be audited first, followed by scattered peripheral high-PI grids, with the audit focus determined by whether the dominant local pattern is low SBG, low DBG, high ES, or a combination of these conditions. Planners can then verify resident needs, street-level conditions, and engineering feasibility before considering any intervention. This sequence uses grid-scale screening to organize local verification (58).

Spatial distributional equity also differs from environmental justice in the broader sense (10). The population-weighted Gini coefficient measures how evenly environmental conditions are distributed relative to the population (21). Keep trajectories represent the spatial activity patterns of users who publicly share records (27). Neither measure shows whether low-income groups, older adults, renters, or people with disabilities receive equivalent environmental benefits (10, 58).

### 4.6 Strengths, limitations, and future research

This study compares static blue-green supply, reconstructed-route DBG, environmental stress, and socioeconomic context within a common 200 m × 200 m grid and the same spatial cross-validation framework (50). It also combines out-of-fold SHAP, domain permutation, interaction analysis, residual testing, robustness checks, and spatial mismatch analysis. Together, these methods examine how environmental domains complement one another, identify nonlinear predictive relationships, and assess their value for descriptive spatial screening (34). The main analytical contribution lies in separating and jointly comparing fixed-grid supply, reconstructed-route exposure, environmental stress, and platform-recorded use within one spatial framework. Machine-learning results provide supplementary predictive evidence.

First, Keep trajectories come from users who choose to record and publicly share their activities, so they cannot represent the daily walking of all residents (27). The number of active Keep users in each grid is unknown, and WAD is therefore not a per-capita walking rate. The 14-day window also cannot capture seasonal, weather-related, or weekday-weekend variation. The conclusions apply only to platform-recorded walking use during the study period. Future work should use longer observation windows, multiple seasons, and multiple data sources.

Second, DBG remains subject to route-reconstruction error and inconsistent spatial support (22, 23). DBG is aggregated by the origin grid of reconstructed routes, whereas WAD accumulates trajectories across the grids they traverse, so the measures are not paired at the individual level. The route audit also covered 4,725 assigned reconstructed routes rather than all publicly shared trajectory records, and reconstructed route lengths differed substantially from the original Keep trajectory lengths. DBG should be treated as an estimate of process exposure along reconstructed routes, not as an exact reconstruction of individual exposure. Point-level GPS data, map matching, and multiple routing engines could improve measurement validity (25, 26). Origin grids may also be represented by different numbers of reconstructed routes, so the precision of grid-level DBG estimates may vary across space.

Third, the predictors do not fully represent the environment in which people decide to walk. Destination mix, street-network connectivity, public transport, air pollution, noise, safety, weather, time of day, and travel purpose were not adequately captured (1, 2, 6, 9). Their omission may help explain the remaining spatial structure in the out-of-fold residuals (50). Future studies should prioritize variables with clear behavioral mechanisms and test the findings across spatial scales, time periods, and cities.

Fourth, the analysis concerns spatial distributional equity relative to population distribution, not environmental justice across social groups (10, 21). PI serves only to screen candidate areas, and its results depend on indicator composition and parameter settings. Adding vulnerability data on age, income, housing stability, and disability status, together with resident surveys or participatory planning, would allow more direct assessment of differences in resource access and environmental burdens among social groups (10, 58).

## 5 Conclusion

This study contributes a descriptive spatial framework for separating fixed-grid blue-green supply, reconstructed-route exposure, environmental stress, and platform-recorded walking use. Its main methodological implication is that static resource supply should not be treated as equivalent to environmental conditions encountered during movement, because the two measures represent different spatial constructs and identify partly different candidate mismatch patterns. Integrating these dimensions therefore supports more differentiated spatial screening and hypothesis generation than reliance on a single blue-green indicator. The framework is intended for descriptive spatial comparison and candidate-area screening, with machine-learning results used as supplementary predictive evidence. The mismatch classes and Priority Index can guide subsequent field audits and local verification. Future research using longer observation periods, true GPS trajectories, additional behavioral and urban-form covariates, and socially disaggregated data is needed to test the generalizability and explanatory value of the framework.

## Data Availability

The original contributions presented in the study are included in the article/supplementary material, further inquiries can be directed to the corresponding author.
